# Acute onset of neovascular age-related macular degeneration after initiation of tirzepatide

**DOI:** 10.1007/s13340-026-00885-7

**Published:** 2026-04-05

**Authors:** Kohzo Takebayashi, Yurina Iemura, Mototaka Yamauchi, Kenji Hara, Takafumi Tsuchiya, Shigeki Machida, Koshi Hashimoto

**Affiliations:** 1 Department of Diabetes, Endocrinology and Hematology, Dokkyo Medical University Saitama Medical Center, 2-1-50, Minamikoshigaya, Saitama 343-8555 Koshigaya, Japan; 2 Department of Ophthalmology, Dokkyo Medical University Saitama Medical Center, 2-1-50, Minamikoshigaya, Saitama 343-8555 Koshigaya, Japan

**Keywords:** Tirzepatide, GIP/GLP-1RA, Subretinal neovascularization, nAMD, Type 2 diabetes

## Abstract

Recent retrospective cohort studies showed that use of glucagon-like peptide-1 (GLP-1) receptor agonists (GLP-1RAs) increased the incident risk of diabetic retinopathy and nonarteritic anterior ischemic optic neuropathy (NAION). Furthermore, it was recently reported that use of GLP-1RAs for more than 6 months increased the risk of neovascular age-related macular degeneration (nAMD) by about twofold, although the association between a gastric inhibitory polypeptide (GIP)/GLP-1 receptor dual agonist (GIP/GLP-1RA) and nAMD is not clearly established. We describe the case of a middle-aged male patient with type 2 diabetes without apparent diabetic retinopathy. Due to poor glycemic control, tirzepatide (a GIP/GLP-1RA) was started instead of sitagliptin (a dipeptidyl peptidase-4 inhibitor). After switching from sitagliptin to tirzepatide, glycemic control rapidly improved, but the patient felt haziness with distortion of the central part of the left eye. A diagnosis of neovascular age-related degeneration (nAMD) was made by ophthalmologists in our hospital. The basis for the possible association of tirzepatide administration with onset of nAMD is unknown. However, clinicians should pay attention to potential visual impairments after achieving acute glycemic control with incretin-related drugs, including tirzepatide.

## Introduction

Incretin-related drugs such as glucagon-like peptide-1 (GLP-1) receptor agonists (GLP-1RAs) and a gastric inhibitory polypeptide (GIP)/GLP-1 receptor dual agonist (GIP/GLP-1RA) are now used worldwide because of their strong effects of lowering blood glucose and body weight [1–4]. Several reports have also shown that use of GLP-1RAs is associated with a significant decrease of cardiovascular events [1–3]. However, particular caution may be needed regarding ophthalmic complications. In the SUSTAIN6 trial, semaglutide (a GLP-1RA) significantly increased diabetic retinopathy complications, including vitreous hemorrhage [2], while a recent retrospective cohort study of patients with type 2 diabetes showed that use of GLP-1RAs modestly increased the incident risk of diabetic retinopathy, although sight-threatening diabetic complications including blindness were somewhat lower in patients treated with GLP-1 RAs [5]. A retrospective cohort study also showed that GLP1-RAs increased onset of nonarteritic anterior ischemic optic neuropathy (NAION) [6]; however, this finding is still controversial [5]. Interestingly, Shor et al. recently reported that use of GLP-1RAs for more than 6 months increased the risk of neovascular age-related macular degeneration (nAMD) by about twofold [7]. However, the association between GIP/GLP-1RA and nAMD is not clearly established.

Herein, we discuss the case of a middle-aged male patient with type 2 diabetes. Due to poor glycemic control, treatment with a weekly subcutaneous injection of tirzepatide (a GIP/GLP-1RA) was started. The patient noticed haziness with distortion of the central part of the left eye about 1 month after initiation of tirzepatide. A diagnosis of nAMD was made mainly based on findings in optical coherence tomography (OCT) and OCT angiography (OCTA) of the retinal pigment epithelium (RPE).

## Case report

A 58-year-old man had been receiving regular treatment for type 2 diabetes at our hospital since the age of 33 years old. His height was 174 cm and body weight was 55 kg at his first visit. He also had hypertension in his 20 s and had started antihypertensive drugs. At 47 years old, he developed intermittent claudication and was diagnosed with arteriosclerosis obliterans based on an ankle brachial index test (right 0.87: left: 1.05) and vascular ultrasound, and administration of aspirin was started. In addition, because of continued dyslipidemia, pitavastatin and ezetimibe were started at 57 years old. He has never smoked. Since 47 years old, he had undergone a regular fundus examination because of early detection of diabetic retinopathy at the ophthalmology department in our hospital, and a status of no diabetic retinopathy with accompanying normal corrected eyesight had been maintained.

Regarding glycemic control, HbA1c had shown repeated fluctuation, but had been stable at about 7% in recent years with adjustment of anti-diabetes drugs. However, for about the last 1.5 years, HbA1c had shown an upward trend to between 8 and 9% despite treatment with glimepiride 1 mg/day, metformin 2000 mg/day, voglibose 0.4 mg/day, and sitagliptin 50 mg/day. About 11 months ago, HbA1c was elevated to 9.2%, and treatment was switched from sitagliptin (a dipeptidyl peptidase-4 inhibitor) to tirzepatide weekly subcutaneous injection (first month, 2.5 mg each week; thereafter, 5 mg each week) to attempt to improve poor glycemic control. After initiation of tirzepatide, HbA1c rapidly improved to 6.2, 6.2, and 6.6% after about 3, 6, and 8 months, respectively. An approximately 5 kg reduction in weight was also noted at 8 months after initiation of tirzepatide.

**Fig. 1:**
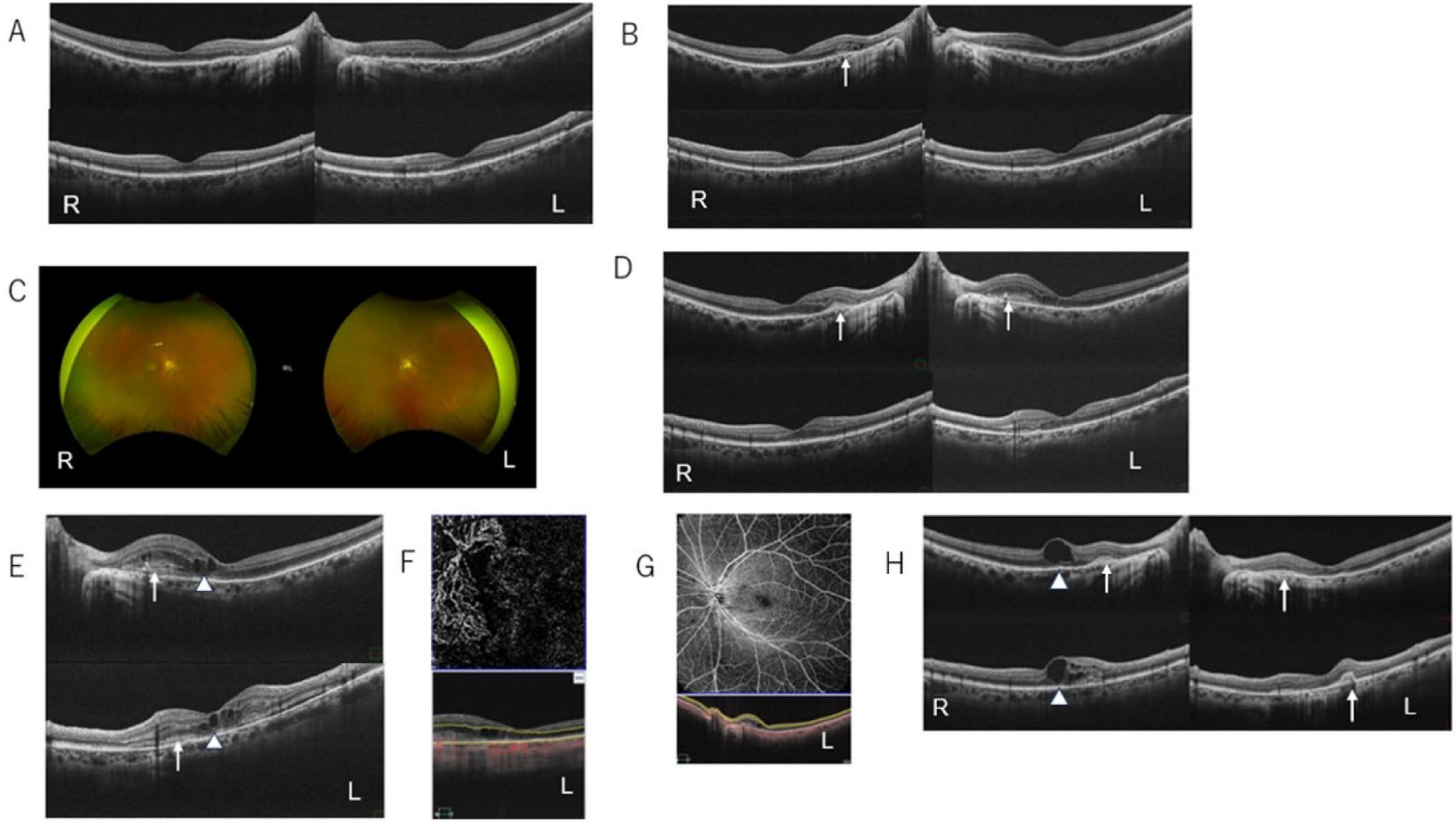
**A** OCT images in the macular area 5 months before initiation of tirzepatide. **B** OCT images in the macular area about one month after initiation of tirzepatide. The arrow shows possible hyperreflected material (SHRM) on the retinal pigment epithelium (RPE). **C** Findings for the ocular fundus about 7 months after initiation of tirzepatide. **D** OCT images in the macular area about 7 months after initiation of tirzepatide. The arrow shows SHRM on the RPE. **E** OCT images in the macular area 3 weeks after the OCT test shown in **D** (about 8 months after initiation of tirzepatide). The arrow shows SHRM on the RPE. The triangle arrow shows intraretinal fluid. **F** En face scan image in OCTA from the outer plexiform layer to the choriocapillaris plate in the macular area 3 weeks after the OCT test shown in **D** (about 8 months after initiation of tirzepatide). **G** En face scan image in OCTA in the superficial layer of the retina in the macular area 3 weeks after the OCT test in D (about 8 months after initiation of tirzepatide). **H** OCT images in the macular area 3 months after the OCT showed in F (about 11 months after initiation of tirzepatide). The arrow shows SHRM on the RPE. The triangle arrow shows intraretinal fluid. R: right eye, L: left eye. For OCT images (**A**, **B**, **D**, **E**, **H**), the upper image shows a horizontal cross section and the lower image shows a vertical cross section

The patient started to feel haziness with distortion of the central part of the left eye one month after initiation of tirzepatide. Five months before tirzepatide was started, there were no abnormal findings in OCT in the macular area in both eyes (Fig. 1A). After 1 month of tirzepatide treatment, there were still basically normal findings in OCT in the macular area, although with slight possible subretinal hyper-reflected material (SHRM) on the RPE in the right eye, but not in the left eye (Fig. 1B). Corrected eyesight was still good (both eyes: 1.2). The OCT findings were similar the next month (i.e., 2 months after initiation of tirzepatide), and careful observation without treatment was continued in the ophthalmology department.

Seven months after initiation of tirzepatide, the patient felt that the haziness in the left eye had worsened, and he visited the ophthalmology department at that time for a regular check-up. Although there were no findings of diabetic retinopathy in the ocular fundus (Fig. 1C), SHRM over the RPE was found in OCT in bilateral eyes, which suggested subretinal neovascularization and vessel bleeding (Fig. 1D). About 3 weeks later, the patient visited the ophthalmology department again because of further exacerbation of haziness in the left eye. SHRM over the RPE became larger and intraretinal fluid had appeared on OCT in the left eye (Fig. 1E). Neovascular vessels was confirmed in an en face scan image from the outer plexiform layer (OPL) to the choriocapillaris plate (CC) (i.e., outer retina/CC:ORCC) in OCTA (Fig. 1F), without abnormal findings in the superficial layer of the retina (Fig. 1G).

These findings suggested the presence of type 2 macular neovascularization (MNV), which indicates invasion of neovascular vessels beneath the retina over the RPE from the choroid. The best-corrected visual acuity was decreased (1.0 right, 0.8 left). Based on these findings, a diagnosis of nAMD was made by ophthalmologists in our hospital, and anti-vascular endothelial growth factor (VEGF) therapy with aflibercept intravitreal injection for the left eye was started 3 weeks later. On the day that nAMD was diagnosed (8 months after the initiation of tirzepatide), tirzepatide was switched back to sitagliptin due to a potential influence of tirzepatide on nAMD, based on a recent report suggesting an association between GLP-1RAs and nAMD [7]. Laboratory data on the day of discontinuation of tirzepatide are shown in Table 1. At that time, there were no apparent neurological findings; and patellar tendon and Achilles tendon reflexes were normal. A switch to insulin therapy is planned if glycemic control becomes poorer. Three months after the diagnosis of nAMD (i.e., after consecutive 3 times administrations of aflibercept: once/a month), the intraretinal fluid in the left eye on OCT vanished, although SHRM over the RPE appears to have become slight lager. On the other hand, in the right eye, intraretinal fluid newly appeared (Fig. 1H).

**Table 1:** Laboratory data on the day of discontinuation of tirzepatide

*Blood counts*	
WBC	6000/µL (3300–8600)
RBC	400 × 10^4^/µL (435–555)
Hb	13.0 g/dL (13.7–16.8)
Ht	38.0% (40.7–50.1)
MCV	95 fl (83.6–98.2)
MCH	32.5 pg (27.5–33.2)
PLT	32.9 × 10^4^/uL (15.8–34.8)
*Chemistries*	
TP	7.2 g/dL (6.6–8.1)
Alb	4.52 g/dL (4.1–5.1)
AST	26 U/L (13–30)
ALT	47 U/L (10–42)
LDH	120 U/L (124–222)
ALP	48 U/L (38–113)
GGT	19 U/L (13–64)
T-Bil	1.63 mg/dL (0.4–1.5)
D-Bil	0.11 mg/dL (0.10–0.30)
Na	137 mEq/L (138–145)
K	5.3 mEq/L (3.6–4.8)
CI	101 mEq/L (101–108)
BUN	12 mg/dL (8–20)
Cr	0.99 mg/dL (0.65–1.07)
eGFR	61.2 mL/min/1.73 m^2^
UA	4.5 mg/dL (3.7–7.8)
Amy	113 U/L (44–132)
TG	73 mg/dL (40–234)
HDL-C	76 mg/dL (38–90)
LDL-C	42 mg/dL (66–163)
*Data related with diabetes*	
Occasional PG	189 mg/dL (78–103)
HbAlc(NGSP)	6.6% (4.9–6.0)
GA	20.4% (11.6–16.0)
*Occasional PG	133 mg/dL (78–103)
*HbAlc(NGSP)	7.0% (4.9–6.0)
*GA	23.0% (11.6–16.0)
*Occasional P-CPR	2.58 ng/mL
*GADAb	< 5.0 U/mL
*UAE	9.5 mg/g.Cr
*Urinalysis*	
protein	(±)
glucose	(−)
blood	(−)
ketone body	(−)

() shows normal values

*WBC* white blood cell count, *RBC* red blood cell count, *Hb* hemoglobin, *Ht* hematocrit, *MCV* mean corpuscular volume, *MCH* mean corpuscular hemoglobin, *PLT* platelet, *TP* total protein, *Alb* albumin, *AST* aspartate transaminase, *ALT* alanine transaminase, *LDH* lactate dehydrogenase, *ALP* alkaline phosphatase, *GGT* gamma-glutamyl transpeptidase, *T-Bil* total bilirubin, *D-Bil* direct bilirubin, *Na* sodium, *K* potassium, *CI* chlorine, *BUN* urea nitrogen, *Cr* creatinine, *eGFR* estimated glomerular filtration rate, *UA* uric acid, *Amy* amylase, *TG* triglyceride, *HDL-C* high density lipoprotein cholesterol, *LDL-C* low density lipoprotein cholesterol, *PG* plasma glucose, *HbAlc* hemoglobin Ale, *GA* glycoalbumin, *P-CPR* plasma C peptide immunoreactivity, *GADAb* glutamic acid decarboxylase antibody, *UAE* urinary albumin excretion

^*^shows the data about 1 month after discontinuation of tirzepatide

## Discussion

A recent retrospective study showed that use of GLP-1RAs for more than 6 months may increase the risk of nAMD [7]. In our case, the patient noted haziness with distortion of the central part of the left eye about one month after initiation of weekly subcutaneous injection of tirzepatide. Subsequently, nAMD was diagnosed based on the presence of SHRM on the RPE in OCT and neovascularization in OCTA. The reason for acute onset of nAMD in this patient is unknown. One possibility may be the acute improvement of glycemic control by tirzepatide. Rapid improvement of glycemic control can cause exacerbation of diabetic retinopathy, so-called “early worsening” of diabetic retinopathy [8], although the exact mechanism is unknown. It is uncertain if this “early worsening” can also occur for nAMD, and this would be of interest to investigate in a large prospective study in patients with diabetes. Another possible reason may be a direct effect of tirzepatide on the retina, since the GLP-1 receptor is expressed in the retina [9]. GLP-1RAs may increase C-X-C motif chemokine ligand 12 (CXCL12) [10], which is associated with VEGF-mediated angiogenesis [11]. CXCL12 has also been shown to attract cells associated with neovascularization of the retina choroid [12]. Therefore, it may be possible that GLP-1RAs are directly associated with onset of nAMD, although this is yet to be shown with clarity.

Tirzepatide is a GIP/GLP-1 dual agonist and acts mainly on the GIP receptor. Thus, the effect of this drug on the GLP-1 receptor is weaker than that of natural GLP-1 or GLP-1RAs such as semaglutide [13]. Therefore, even if the above hypothesis (a direct effect of a GLP-1RA on onset of nAMD) is correct, there is a need to examine if tirzepatide can influence the GLP-1 receptor in the retina. However, the GIP receptor has also been found to be expressed in retina in rat and mouse models, although its role is uncertain [14, 15]. Thus, it will be of interest to investigate whether a pure GIP agonist can influence neovascularization of the retina choroid. In our case, anti-VEGF therapy was started and tirzepatide was switched back to sitagliptin. However, the progression of nAMD still had continued 3 months after the suspension of tirzepatide. Careful long-term observation is needed to evaluate whether these treatments contribute to improvement of nAMD.

Recently, the relationship between GLP-1RAs and NAION has been suggested [6]. Although NAION and AMD (including nAMD) is basically different condition, NAION was significantly associated with AMD in a retrospective cohort study [16]. The most accepted cause of NAION is based on ischemia of the anterior optic nerve resulting from hypoperfusion of the short posterior ciliary arteries (SPCAs)[17, 18]. On the other hand, although the detailed mechanisms of the onset of nAMD are not completely elucidated yet, the decrease of choroidal blood flow may contribute to the development of early AMD at least partially [19, 20]. The choroidal capillaries circulation is supplied by SPCAs [19]. Therefore, although there is no apparent evidence at the current time for overlapping mechanisms between the development of NAION and nAMD, it may be interesting to consider the involvement from impaired ocular blood flow (probably based on hypoperfusion of SPCAs) as a possible common mechanism of both conditions, and to investigate if GLP-1RAs or GIP/GLP-1RA (tirzepatide) can affect directly or indirectly the perfusion of SPCAs and choroidal capillaries.

In this case, 5 months before the initiation of tirzepatide, the OCT findings was normal and optical clinical findings became apparent after the initiation of tirzepatide. However, the possibility that the early change of nAMD, in which OCT findings and clinical symptoms may not be emerged yet, had already been present before using tirzepatide cannot be completely excluded.

Smoking is one of the most important risk factors of AMD [21]. However, the patient in this case has never smoked before. This should be noted as the important characteristics of this case.

In conclusion, we encountered a patient with type 2 diabetes who received tirzepatide due to poor glycemic control. After initiation of tirzepatide, glycemic control acutely improved, but nAMD developed rapidly. The association of onset of nAMD with tirzepatide in this case is uncertain. However, the case suggests that clinicians should pay attention to potential visual impairments after acute improvement of glycemic control by incretin-related drugs, including tirzepatide.
